# Alzheimer’s Disease Drug Development Pipeline 2020

**DOI:** 10.14283/jpad.2020.12

**Published:** 2020

**Authors:** M.N. Sabbagh

**Affiliations:** Cleveland Clinic Lou Ruvo Center for Brain Health, 888 W. Bonneville Ave, Las Vegas, NV 89106

On March 21, 2019, the aducanumab development team conducting the EMERGE and ENGAGE studies announced that the trials had met a pre-specified futility endpoint (1). The consequences of that announcement were far reaching, as many companies that had invested heavily in targeting amyloid were left to consider if targeting amyloid still remained a viable strategy for developing effective Alzheimer’s disease (AD) therapeutics. Following that announcement, the remaining BACE inhibitor trials announced safety concerns or efficacy concerns effectively ending elenbecestat, umibecestat, lanabecestat, atabecestat, and verubecestat as possible treatments for AD (2). Given that gamma secretase inhibitors and modulators had failed in the past, altering or halting production of amyloid appears to no longer offer viable therapeutic benefits moving forward. To further complicate this already troubling situation, the TMS/cognitive stimulation treatment combination (3) has not received FDA approval.

On October 22, 2019, the sponsor of EMERGE announced that their data was indeed positive for demonstrating clinical efficacy and anticipated filing in 2020 for a clinical indication with the FDA (4). Overnight, the field was re-invigorated with the possibility of an approvable drug, and research investigating anti-amyloid methods was encouraged. The mABs Gantenerumab and BAN2401 have launched their phase III clinical trials with results expected in 2022 or 2023. The mAB programs consider increasing doses and targeting oligomers a potentially appropriate strategy.

The rise and fall and rise again of anti-amyloid methods have opened the way for evaluating non-amyloid-centric methods into 2020 and beyond. One expansive, emergent concept gaining traction is segmentation in the therapeutic space. This concept is appealing because the symptoms and pathology of AD are diverse. Segmentation targets a specific symptom or subset of the disease rather than treating all patients with the disease. Prominent examples of this include the Alz 801 molecule that is entering phase III studies which is randomizing Apo E 4 homozygotes exclusively. Another example is pimavenserin, a selective serotonin inverse agonist (SSIA) preferentially targeting 5-HT2A receptors that are thought to play an important role in dementia-related psychosis (5). The sponsor announced positive results from the HARMONY study for the treatment of dementia related psychosis; a target symptom. Similarly, brexpiprazole is in phase III studies to treat agitation in dementia; a target symptom. The orexin antagonist suvorexant targets insomnia, a common symptom among AD patients, and has shown potential therapeutic benefits for mild to moderate AD. This drug has entered Phase III trials. More drugs being developed that are symptoms or subset specific.

Another trend into 2020 and beyond is the emergence of non-amyloid approaches. Prominent among these are the mABs targeting tau isoforms in an effort the block the prionosis spread of p-tau from neuron to neuron. Many companies have phase II clinical trials exploring the mABs targeting tau but there are drugs in development targeting tau that are not mABs. Non-amyloid methods also include the gingipain inhibitor (Corteyme -COR388). Gingipains are byproducts of oral bacteria p. gingivalis and have been shown to be neurotoxic. Other strategies in phase III clinical trials include anti-hypertensives, tyrosine kinase inhibitiors, AGB101 (a proprietary levetiracetam formulation that is dosed to target hippocampal hyperactivity), Anavex 2–73 (a molecule that binds sigma 1 and targets protein misfolding), and plasma exchange (AMBAR program), and the Hepatocyte Growth Factor agonist NDX-1017. The AMBAR program contains amyloid directed elements, but the premise that infusion of albumin to reduce oxidation is non-amyloid. Thus, the plasma exchange-albumin infusion, which shows promising results in moderate AD dementia, might contain multiple mechanisms of action. The future of the RAGE inhibitor azeliragon seems uncertain.

The future of Alzheimer’s treatment might be a multidrug, multi-modal approach akin to chemotherapy. Still undecided is how to combine potential drug treatments and how to select the appropriate subgroups and in what order they should be selected. These trends clearly include segmentation and non-amyloid directed methods and will fundamentally alter the therapeutic landscape and force us as researchers and clinicians alike to think expansively.
